# Concurrent Deficiency of Factor V and Factor VIII in a Pediatric Patient: A Case Report

**DOI:** 10.7759/cureus.45663

**Published:** 2023-09-21

**Authors:** Tanzeel U Rahman, Noman Salih, Nasar Rashid, Maaz Ahmad, Asad Khan, Zainab Yousufi

**Affiliations:** 1 Paediatrics, Hayatabad Medical Complex Peshawar, peshawar, PAK; 2 General Internal Medicine, Hayatabad Medical Complex Peshawar, Peshawar, PAK; 3 Paediatrics and Child Health, Hayatabad Medical Complex Peshawar, Peshawar, PAK

**Keywords:** bruises, hematuria, epistaxis, factor v and viii deficiency, haematological

## Abstract

This case report delves into an uncommon coagulopathy recognized as factor V and VIII deficiency (F5F8D), which follows an autosomal recessive inheritance pattern. The focal point of this study is a five-year-old Asian female who was initially presented with complaints of hematuria, epistaxis, and bruises all over the body. Comprehensive haematological and coagulation profiling unveiled indicators such as diminished haemoglobin levels and prolonged activated partial thromboplastin time (aPTT), prothrombin time (PT), and international normalized ratio (INR). Subsequent factor assays demonstrated noteworthy reductions in both factor V and factor VIII activities, unequivocally confirming the existence of a concurrent deficiency in these crucial factors. Notably, patients exhibiting elongated INR, PT, and aPTT values necessitate a comprehensive assessment for potential combined deficits in factors V and VIII when formulating a differential diagnosis. In cases where substantial bleeding manifestations are evident during the patient's presentation, it is prudent to exercise judicious medical management strategies.

## Introduction

An uncommon autosomal recessive constitutional hematological disorder, known as combined factor V and factor VIII deficiency (F5F8D), was initially documented in 1954 by Oeri et al. [1]. The prevalence of this condition differs across geographical regions, with Middle Eastern countries reporting an estimated prevalence of 1 in 100,000, while in other global regions, it is much rarer at 1 in 1 million. This disorder predominantly manifests in Asia and the Mediterranean region, particularly in Middle Eastern nations. The condition's manifestation requires a heterozygous genotyping pattern in the parents of affected individuals, indicating a higher occurrence in areas with a history of consanguineous marriages.

Clinically, patients with combined factor V and factor VIII deficiency often exhibit a propensity to bleed, with varying degrees of severity ranging from mild to moderate. Clinical manifestations encompass soft tissue bleeding, gum bleeding, menorrhagia, easy bruising, epistaxis, and post-dental surgery bleeding [2].

Diagnostic evaluation reveals noteworthy delays in the international normalized ratio (INR) and activated partial thromboplastin time (aPTT), while bleeding time and platelet count remain within normal ranges. Corrected prothrombin time (PT) and aPTT through mixing studies have been observed. Further factor assays are recommended for a comprehensive diagnosis. The detailed genetic analysis offers the potential to elucidate the precise gene mutation underlying this condition.

In the management of bleeding episodes following surgical interventions, successful interventions include the administration of one-deamino-8-D-arginine vasopressin (DDAVP) and fresh frozen plasma (FFP) [3].

## Case presentation

We present the case of a five-year-old South Asian child who was admitted to the emergency department with a constellation of symptoms. These included hematuria, epistaxis, and bruises all over the body for more than two months. Upon examination, the patient exhibited pallor, evident multiple bruises on the body, and observable blood traces within the nasal passages. Additionally, the urine displayed a subtle reddish hue. The clinical assessment revealed no other significant findings apart from an elevated heart rate, while blood pressure and oxygen saturation levels remained within the normative range.

The patient's medical history was notable, encompassing a proclivity for recurrent nosebleeds, widespread bruising, and recurring hematuria. No antecedents of musculoskeletal or joint haemorrhages were reported. It is pertinent to note that the patient's parents were consanguineous, sharing close familial ties. Of note, the patient had a younger sibling who presented with similar symptoms.

Laboratory investigations were performed (Table 1). Aside from microcytic hypochromic anaemia, the haematological assessments indicated normal platelet counts, unperturbed peripheral smears, PT of 25.6 seconds, INR of 2.46, and aPTT of 103.3 seconds. The direct and indirect Coombs tests were negative as well. Further testing involving mixing studies rectified the aberrant PT, INR, and aPTT values.

**Table 1:** Baseline investigations Hb: hemoglobin; WBC: white blood count; APTT: activated partial thromboplastin time; PT: prothrombin time INR: international normalized ratio; PLT: platelet; Fe: Iron; Na: sodium; K: potassium; ALP: alkaline phosphatase

Test	Absolute value	Reference value
WBC (µL)	8000	4000–11,000
Hb (g/dl)	7.1	12.1-15.1
PLT (µL)	393,000	150,000–400,000
PT (sec)	25.6	12
Bleeding time (minutes)	3	2–9
Clotting time (minutes)	Greater than 15	2–8
aPTT (sec)	103.3	28
INR	2.46	1
Fe (mcg/dl)	28	50–170
Na (mEq/L)	144	135–145
K (mEq/L)	4.3	3.5–5.2
ALP (IU/L)	55	44–147
Uric Acid (mg/dl)	4.8	3.5–.2

In light of the persistently elevated PT, aPTT, and INR, the patient received prophylactic treatment with fresh frozen plasma and packed red blood cells (PRBCs) in order to stop bleeding, improve her red blood cell count, and relieve her symptoms. Additionally, the administration of tranexamic acid and vitamin K was carried out to decrease her bleeding episodes and correct any vitamin deficiency-related bleeding. The patient did not experience any bleeding episodes.

In order to conduct thorough factor assays and accurately diagnose the patient, they were sent to an advanced care facility. The factor assays conducted at a reliable local laboratory yielded the results shown in Table 2, which indicated reduced activity of both factors V and VIII. Correction studies were performed for prothrombin time, which yielded a value of 10.9 seconds with normal plasma, a value of 11.6 seconds with adsorbed plasma, a value greater than 100 with normal serum, and a value of 28 with aged plasma, confirming the diagnosis of combined factor deficiency. Consequently, the patient was counselled regarding regular follow-up visits, given the coexistence of deficiencies in factors V and VIII. Recommendations included the provision of factor VIII supplementation and two units of FFP as necessary in instances of bleeding or surgical interventions. The patient has been followed up on a monthly basis and is receiving multivitamins and iron supplements on a regular basis. Also, we are monitoring his status with CBC, PT/aPTT, and INR, and he is doing well.

**Table 2:** Coagulation profile F: factor; P.S: protein S; P.C: protein C

Test	Absolute value	Reference value
F II level	85%	80–120
F V level	14%	80–120
F VII level	65%	65–150
F VIII level	4%	80–120
F IX level	73%	70–140
F X level	78%	80–120
F XII level	75%	80–120
D-dimer	1.47 µg/mL FEU	0–0.5
Fibrinogen level	6.54 g/ L	1.4–4.4
Anti-thrombin III	104%	80–120
P. S function	0.6–1.40	0.75 IU/mL
P. C function	0.5–1.24	0.92 IU/mL

## Discussion

Congenital mixed factor V and factor VIII deficiency represents a rare autosomal recessive bleeding disorder that exhibits a higher prevalence in regions characterized by consanguineous marriages. The most prevalent form of mixed plasma coagulation factor deficiencies is the deficiency of both factor V and factor VIII. This condition has been documented in families across diverse geographic regions, including the Middle East, North America, Europe, the Far East, and South Asia, with the highest incidence observed among individuals of Middle Eastern descent [2].

The aetiology of this disorder is attributed to mutations in the LMAN1 gene (located on chromosome 18; 18q21) or the MCFD2 gene (located on chromosome 2; 2p21). These mutations disrupt the proper functioning of the ERGIC-53/MCFD2 protein complex, responsible for the transportation of coagulation factors V and VIII from the endoplasmic reticulum to the Golgi apparatus. Notably, null mutations in LMAN1 account for approximately 70% of cases, while MCFD2 mutations, comprising both null and missense variations, are responsible for about 30% of cases [3,4]. Regrettably, our patient was not subjected to genetic screening for these mutations, attributed to limitations in available resources.

The diagnosis of this condition is achieved through a comprehensive screening assay involving factor levels. The standard range for factor V and factor VIII levels is between 80% and 120%. Although most individuals with F5F8D exhibit levels between 6% and 32%, levels may range from 1.5% to 45%. In our case, the patient's factor V and factor VIII levels were measured at 14% and 4%, respectively. Furthermore, activated partial thromboplastin time and prothrombin time are typically investigated to exclude other potential diagnoses [2,3]. We decided to report this case in South Asia after studying the relevant literature. Our research revealed that the incidence of this condition is gradually rising in the region because of consanguineous marriages [5]. Our aim is to ensure that healthcare professionals in the area are well-informed about it.

The concomitant reduction in plasma levels of factor V and factor VIII gives rise to mild to moderate symptoms within the afflicted population. Although the manifestation of bleeding symptoms can demonstrate variability, they frequently parallel those encountered in isolated deficiencies of either factor V or factor VIII. Notable clinical presentations encompass episodes of epistaxis, menorrhagia, susceptibility to ecchymosis, bleeding subsequent to trauma or surgical procedures, and, to a lesser extent, occurrences of haemarthrosis and muscular hematomas [4,5]. Our patient's medical history encompasses instances of epistaxis, hematuria, and widespread bruises over the body.

Management of combined F5F8D typically involves factor replacement therapy, with prophylaxis being considered in cases of recurrent haemarthrosis or intramuscular haemorrhage. The Hemophilia Centre Doctors' Organization in the United Kingdom recommends a treatment regimen utilizing FFP, which provides concentrated factor V and factor VIII. For factor VIII replacement, specific factor concentrations from either plasma-derived or recombinant sources are preferred. Studies have demonstrated the efficacy of FFP-based regimens in controlling bleeding during dental procedures, circumcisions, and labour induction. Desmopressin can also be utilized in select scenarios for managing minor bleeding episodes [5,6]. The study by Sallah et al. found that in CF5F8D patients, DDAVP can raise FVIII levels but has no impact on the use of plasma exchange in hereditary deficiency of factor V and factor VIII FV plasma concentration. These findings imply that FVIII concentrates may serve as an alternative to DDAVP in CF5F8D patients [7]. Prognostically, milder instances of this condition generally exhibit a favourable outlook, while individuals with more severe forms require specialized care within a hospital setting [3].

## Conclusions

Nations situated within South Asia, the Middle East, and the Mediterranean region, characterized by prevalent consanguineous unions, present elevated incidences of combined factor V and factor VIII insufficiency. In instances where patients demonstrate prolonged PT and aPTT, alongside the fulfilment of corresponding epidemiological benchmarks, prudent consideration should be given to the possibility of this medical disorder. The management of recurrent and severe hemorrhagic episodes necessitates a structured approach, primarily involving the systematic administration of FFP and concentrated factors.
